# Two-Tailed Dogs, Social Unrest and COVID-19 Vaccination: Politics, Hesitancy and Vaccine Choice in Hungary and Thailand

**DOI:** 10.3390/vaccines10050789

**Published:** 2022-05-16

**Authors:** Robin Goodwin, Lan Anh Nguyen Luu, Juthatip Wiwattanapantuwong, Mónika Kovács, Panrapee Suttiwan, Yafit Levin

**Affiliations:** 1 Faculty of Psychology, University of Warwick, Coventry CV4 7AL, UK; robin.goodwin@warwick.ac.uk; 2 Institute of Intercultural Psychology and Education, Eötvös Loránd University, 1075 Budapest, Hungary; lananh@ppk.elte.hu (L.A.N.L.); kovacs.monika@ppk.elte.hu (M.K.); 3 Faculty of Psychology, Chulalongkorn University, Bangkok 10330, Thailand; cpanrapee@yahoo.com; 4 Department of Social Work and Education, Ariel University, Ariel 4076414, Israel; yafitl@ariel.ac.il

**Keywords:** vaccines, politics, Hungary, Thailand, culture

## Abstract

Background: A long tradition of research has shown an association between political orientation and vaccine uptake. However, we know little about political preferences and the choice of specific vaccines. Methods: We conducted two national surveys, in Hungary (Study 1, online, *n* = 1130) and Thailand (Study 2, on the street survey: *n* = 1052), testing associations between political allegiance, trust in government, vaccine willingness, and vaccine choice. Results: In Hungary, those supporting the government or on the political right were more willing to be vaccinated, with this association strongest for government approved vaccines. These respondents were also more likely to accept Chinese and Russian vaccines and reject the Moderna vaccine. In Thailand, vaccinated respondents reported greater trust in the government, with preference for AstraZeneca associated with support for pro-government political parties and preference for Pfizer with anti-government attitudes. Conclusions: Vaccine campaigns need to recognise the role of political loyalties not only in vaccine willingness, but in vaccine choice, especially given the mixing of vaccines across doses.

## 1. Introduction

A long tradition of research has demonstrated the association between political partisanship and behaviour during pandemics [1,2]. The politicisation of pandemic responses has intensified during COVID-19 [3], with vaccine uptake also showing a significant political dimension [4,5,6]. In the US, Republicans expressed less willingness to vaccinate [7,8]. In France, those with no political party attachments were more likely to refuse a vaccine [5], as were those who were members of more non-institutional parties (more radical parties or political movements). A political dimension has been particularly evident where populations have had an element of vaccine choice. In Brazil, a national survey of 2771 adults found supporters of President Bolsonaro were less likely to vaccinate than those who opposed him and were more likely to reject a vaccine developed in China [9].

This politicisation of COVID-19 vaccination has been clearly evident in two countries relatively under-studied during the pandemic. In the decade before the COVID-19 pandemic, Hungary underwent considerable political turmoil, with an increase in authoritarian social control and nationalism under Victor Orban [10]. During the second wave of Coronavirus (winter 2020), the Hungarian government entered talks with Russian and Chinese authorities to try to obtain vaccines outside of the EU [11]. An important distinction was made between the range of vaccines approved only by the government and those accepted by the European Union [12,13]. The former included Chinese and Russian vaccines at the time not approved by the European Medical Association (EMA), the latter the EMA-approved Pfizer, AstraZeneca, Johnson and Johnson, and Moderna vaccines. A cross-cultural analysis conducted across Central and East Europe (April 2021) found greater support for a Chinese vaccine in Hungary than any of the other nine nations surveyed, although the preference in Hungary was still for a Western European or American vaccine [14], as was common across Eastern Europe [15]. In Thailand, the pandemic also coincided with a period of considerable political unrest. After years of contestation between supporters of the red and yellow shirts [16], a series of large anti-government demonstrations in early 2020 served to polarise society, with many different elite groups helping drive this process [17]. Government and royal support for a small bioscience company, with no history of vaccine manufacturing, contributed towards vaccine scepticism, a delayed rollout, and raised questions about the political stance of AstraZeneca, produced under license in Thailand at the time [18,19,20]. At the same time, there were media reports of widespread demands for mRNA vaccines, perceived by many Thais as more effective in preventing disease [18,21,22].

### 1.1. Theoretical Perspective

In this paper, we consider associations between political allegiance, vaccine willingness, and choice of vaccine(s). Several potentially related theoretical perspectives have argued that political allegiances may sharpen during a time of pandemic, reflecting the view that health behaviours do not occur in a vacuum [23]. Social identities, people’s “sense of selfhood defined by their group memberships” [24] (p. 202), provide a framework for meaning and actions during a pandemic, including their willingness to undertake vaccination [25]. While common, national and shared identities may emerge early on in a crisis and encourage disease-preventive behaviours [26], polarisation can soon emerge [24], with political factors having different impacts in different cultures [27]. Relatedly, terror management theory suggests that, when mortality threat is high, first, people seek to consciously deny or avoid the threat or ameliorate this through appropriate preventive behaviours (e.g., handwashing). However, over time and less consciously, they seek to affirm their cultural worldviews as a form of distal defence [28]. This then leads to disparate, partisan attitudes and behaviours [29]. At this time, trust in government is particularly important for influencing health behaviours [24] including vaccination [6,30,31].

While considerable research has considered the individual and group level determinants of vaccine rejection or hesitancy, less work has considered perceptions of specific vaccines [32]. Political polarities are also likely when considering willingness to take vaccinations originating in different countries. From a social representations perspective, we anchor our views of these vaccines, and their countries of origin, in images and metaphors that exist in “everyday knowledge” around cultures and diseases [33,34]. Specific countries of manufacture, and particular vaccine manufacturers, are likely to be associated with both the efficacy and safety of a vaccine. Beliefs about the influence of foreign powers and specific vaccines has made not only vaccine willingness but also vaccine choice subject to a plethora of historical and cultural influences, with mRNA vaccines associated with American influence [35] and Sinovac with Chinese [22,36]. Media, both traditional (legacy) and social, helps amplify these representations [37] and has played an important role in vaccine decision making [6,38]. This should not be seen as the simple propagation of false beliefs but as reflective of other underlying concerns (e.g., about 5G mobile technology) [6] and the treatment of particular cultural groups during previous vaccination campaigns [39]. Such representations also often fit into wider political meta-perspectives, such as a divide between the East and West (evident within Hungarian politics) and high levels of distrust amongst some communities (e.g., Roma in Hungary [40]). These representations of vaccines intersect, and sometimes conflict with, governmental policies and the sheer availability of certain vaccines. Economic factors, such as the availability of specific vaccines in private hospitals in Thailand [41] and the reselling of vaccine slots [42], have further confounded the situation.

### 1.2. Our Studies

Our studies provide novel insights into the association of political preferences, trust, and both vaccine willingness and vaccine choice in two relatively understudied and politically riven countries. Our first study examines vaccine uptake and willingness during the early vaccination rollout in Hungary. At the time of our Hungarian data collection (spring 2021), several Hungarian political opposition parties had united to provide a combined front for the 2022 election as a challenge to the ruling FIDESZ party. We consider associations between vaccine willingness and choice by assessing political allegiances in three ways, comparing: (i) supporters of the ruling FIDESZ party vs. advocates of other political parties (ranging from Socialist MSZP Party to the right-wing Jobbik and satirical Two-Tailed Dogs Party) [10], (ii) preferences for parties broadly aligned to different positions of the party-political spectrum (left, centre, or right-wing), (iii) self-rated political affiliations, scored on a percentage scale from left to right. As vaccine intention has been found to be more positive amongst men, those over 60 years of age, and the more educated, in both Hungary [14] and Thailand [43], we control for these demographic factors in our analyses, alongside participant’s health status. We anticipated that those who declared themselves sympathetic towards the current (right-wing) government in Hungary, and those in general who indicate themselves to be on the political right, will be more willing to vaccinate with a vaccine approved by the government, and will show greater preference towards Chinese and Russian vaccines, than those who oppose the government, or support parties on the political left/centre.

Our second study considers vaccine willingness and choice at a time when most respondents had received their initial vaccinations but were eligible for booster vaccines. In Thailand, Sinovac (Coronovac) was originally the main vaccine offered against COVID-19, but questions over its efficacy led to recommendations to combine a Chinese and Western vaccine, with the locally made AstraZeneca (Vaxzevria) vaccine suggested for a second dose [44]. However, the use of both these vaccines was associated with vaccine scepticism [45], with evidence of stronger preference for Pfizer. We expect this preference to be reflected in our data, with our emphasis now on the third (booster) vaccine. We anticipate trust in government to be positively associated with both vaccine willingness and acceptance of the initially recommended vaccines (Sinovac and AstraZeneca). Political allegiances could be broadly categorized into two camps, pro-government (Palang Pracharat, the Democratic Party and Phum Jai Thai) vs. opposition parties (Pheu Thai and Future Forward/Moving Forward). We expect vaccine willingness and acceptance of the vaccines used in the early rollout to be greater amongst those who support parties positive towards the government. As elsewhere, we anticipate vaccine uptake to be greater amongst older populations [18,43,46], men [43,46], and more educated populations [43,46].

## 2. Materials and Methods. Study 1: Vaccine Uptake and Vaccine Choice in the Unvaccinated in Hungary

### 2.1. Methods

#### 2.1.1. Participants

A particular challenge for our work was the rapid acceleration of the vaccine rollout in Hungary in the spring of 2021. Given that we were studying vaccine willingness and choice, it was important for us to collect data from those yet to be vaccinated. We conducted an online survey between 8 and 16 April 2021. Participants responded to a request from an online national panel survey company (Medián Opinion and Market Research), with a target sample size of 1000, in line with previous studies examining vaccine willingness in smaller-sized countries [47] as well as common practice and opportunities for data collection within that country. Reminders for survey completion were sent first by age, then by sex and region, in order to generate a representative sample by sex, age, and geographical distribution (see Table 1, which provides data on the education, region and political affiliations of respondents). Of 1450 beginning the survey, 1130 (79%) met inclusion criteria (were aged over 18 or over, passed a time verification check, and had not already been vaccinated) and fully completed the survey. Participants had a mean age 50.53 (SD 15.92, median 52); 618 (54.7%) were female, with the largest response group having completed secondary education but not further studies. Surveys took on average 16 min to complete.

#### 2.1.2. Measures

Participants indicated their sex, age, and education, categorised into highest educational achievement (Primary, Secondary, College/BA, Masters, Doctoral). Respondents indicated if they had been personally diagnosed with COVID-19, knew someone previously seriously ill from COVID-19, whether they were in one of the CDC risk groups for COVID-19, were pregnant or planning pregnancy (all yes/no)) and their self-rated health, on a four-point scale (bad to very good). Voting intention was captured by asking *If there is an election at the weekend which party would you vote for?* Respondents answered in free format, subsequently coded by the second fand fourth authors into the major political groupings. This was then coded into (i) ruling FIDESZ vs. other parties and (ii) political orientation of the party selected (centre/left vs. right-wing parties). We also asked *Where would you place yourself politically on a percentage scale*? (from very left (0) to very right (100)).

We examined both willingness to accept a vaccine approved by the government and, separately, the subset of vaccines approved by the EMA (*I would be willing to accept a vaccine approved safe and effective by the government* (*by the European Medical Agency*)). For both indicators, we used 5-point scales (*completely unwilling* to *completely willing*), using the full scale, rather than collapsing response categories [48], in order to capture the full association between vaccination willingness and vaccine behaviour [8]. For vaccine choice, we asked respondents *If you could choose the vaccine type, which one would you choose (you can choose more than one)*. Participants were then given the option of ticking one or more from a list of six vaccines (Johnson & Johnson, Moderna, AstraZeneca, Pfizer, Sinopharm, Sputnik), all of which were widely available for us in Hungary at the time of our survey [49].

#### 2.1.3. Statistical Analysis

Competitive path analyses, controlling for demographic and health variables, examined willingness to be vaccinated by vaccines approved only by the government and those approved by the EMA, comparing the predictive validity of the two political groupings (FIDESZ supporters vs. advocates of other parties; supporters of broadly left or central parties vs. right-wing parties) as well as self-rated political position. Analyses were conducted using structural equations modelling (SEM) with Mplus8.1. The robust estimation method was used to estimate maximum likelihood of model fit [50]. The adequacy of model fit was assessed through multiple indices, with the ratio of the Satorra–Bentler χ² to degrees of freedom also used, with ratios of 2.0 or less indicating the absence of significant unexplained error. The non-normed fit index (NNFI) and comparative fit index (CFI) used values of 95 or higher as indicators of a good fit [51]. Root mean square error of approximation (RMSEA) was used to consider the error of approximation in the population and the difference between the proposed model and actual variances and covariances in the data, with values <06 preferred [51].

To test associations between vaccine choices and political groupings, we used political groupings as predictors with vaccine choices (binary scored) as dependent variables. For the political groupings we used two logistic regressions; for the continuous score of political tendency (from 0–100) we used linear regression. For each analysis, again, we controlled for the demographic/health variables. Raw data and question items are deposited at https://osf.io/65gzp/ (accessed on 10 April 2022).

### 2.2. Results

All but one respondent indicated their political position on a percentage scale (*n* = 1130; M position = 50.44, SD 20.28). Of those who indicated a political party preference 173 asserted support for the ruling FIDESZ party, 468 for other parties, with the remainder either responding indicating their refusal to say (50), no preference (78) or they were unsure (136). We classed as right-wing those who supported Christian Democrats KDNP, FIDESZ, Jobbik, or our Homeland Party (*n* = 341), and those who supported centre-left or left-wing, Greens (LMP), Democratic Coalition, Momentum, the Socialist MSZP, and the Two Tailed Dogs Party (*n* = 300). As a sensitivity analysis, we compared the political groupings on the self-perceived political percentage scale. FIDESZ supporters were more right-wing than those who opposed this party (FIDESZ (69.34%/100) vs. others (44.30%) (t (342) = 13.35 *p* = 0.001), with this distinction even sharper between those on the broadly left/centre parties vs. right-wing parties (right-wing vs. centre/left parties M = 62.7% vs. M = 37.79%) (t(627) = 15.15 *p* = 0.001)). We report results from each path analysis below and in Table 2. This presents estimates of the path analyses examining the contribution of political preference on willingness to vaccinate with a government-only approved vaccine, or an EMA approved vaccine, allowing for demographic factors, COVID experience, and self-rated health. In Table 2, we provide three different ways of identifying political preference: (1) supporting the government party (FIDESZ) vs. advocates of other parties; (2) supporting broadly left or central parties vs. right-wing parties, and (3) identifying oneself on a political percentage scale ranging from left to right.

### 2.3. Willingness to Vaccinate Contrasting FIDESZ vs. Other Parties

Path analysis showed satisfactory fit to the proposed model (χ2 [20] = 57.61; *p* < 0.001; χ2/df ratio = 2.88; CFI = 0.98; NNFI = 0.99, RMSEA = 0.02 [C.I. = 0.00, 0.08]). The total variance explained was 16% for willingness for a government-only approved vaccine and 9% for an EMA approved vaccine.

Support for FIDESZ was significantly associated with both willingness to vaccinate with the government-only approved vaccine and the EMA approved vaccine. Age was positively associated with greater willingness to vaccinate with the EMA-approved vaccines. Gender (male) was significantly associated with willingness to vaccinate with the government-only approved vaccine. Higher education levels were associated with both higher willingness to vaccinate with the government-only approved vaccine and EMA approved vaccines. Risk group membership was associated with a greater willingness to vaccinate with the EMA approved vaccine but not with the government-only approved vaccine. Participants diagnosed as positive for COVID-19 were not more willing to vaccinate. However, participants who had a significant other sick from COVID-19 were more likely to accept both the government-only and EMA approved vaccines. Self-rated health was positively associated with willingness to vaccinate with the EMA approved vaccine, but not the government-only approved vaccine.

### 2.4. Predicting Willingness to Vaccinate by the Right vs. Centre/Left Political Orientation

Analyses also revealed satisfactory fit (χ2 [20] = 76.46; *p* < 0.001; χ2/df ratio = 3.82; CFI = 0.96; NNFI = 0.99; RMSEA = 0.05 [C.I. = 0.038, 0.062]), (9% total variance for government-only approved vaccine, 9% for EMA approved vaccine). Supporting a right-wing party was positively associated with willingness to vaccinate with the government-only approved vaccine but not with the EMA approved vaccine. Other covariates were related to vaccine willingness in a manner similar to the previous model (above).

### 2.5. Predicting Willingness to Vaccinate by the Political Tendency (Very Left to Very Right, from 0–100)

Analyses revealed a satisfactory fit to the proposed model (χ2 [20] = 76.35; *p* < 0.001; χ2/df ratio = 3.82; CFI = 0.95; NNFI = 0.94; RMSEA = 0.05 [C.I. = 0.038, 0.062]) (8% variance for willingness to the accept government-only approved vaccine, 9% for EMA approved vaccine). Right-wing tendency was significantly positively associated with willingness to vaccinate with the government-only approved vaccine but not with the EMA approved vaccine. Other covariates were similar to the previous models (above).

### 2.6. Vaccine Choice and Politics

Across the sample, Pfizer was the most preferred vaccine choice (by 60.9%) followed by Moderna (28.5%), Sputnik (22.8%), Johnson & Johnson (21.8%), Sinopharm (11.7%), and AstraZeneca (9.2%). Logistic regressions (Table 3) examined the choice of individual vaccine by political affiliation, controlling for demographics, COVID experience, and self-rated health. As before, we used three different indices of political affiliation. Our results showed that the selection of Moderna and Pfizer was associated with left-wing political tendencies, both when political parties are categorized as right vs. left and when political position is treated on a continuous range. However, of these two vaccines, only Moderna was negatively associated with political party when using the comparison between FIDESZ and other parties. Choosing Sinopharm or Sputnik was associated with supporting the government party or more right-wing tendencies for each political grouping.

### 2.7. Discussion of Study 1

In our data, those who supported the government party, or were supporters of broadly right-wing parties or on the political right, were more willing to accept a government-only approved vaccine. In addition, the vaccines preferred by our Hungarian respondents echoed the broader political debate in Hungary about the EU and wider East-Western loyalties, with those supporting FIDESZ and those on the political right more accepting of the Sputnik and Sinopharm vaccines. As with other surveys in Hungary [14], preference was greatest for Pfizer and Moderna vaccines, while reluctance across the sample to accept the AstraZeneca vaccine may have reflected contemporaneous concerns about blood clotting [52]. At the close of our survey, Hungary had reported 742,198 cases and 24,762 deaths in a total population of 9.64 million, placing it amongst the highest death rates in the world. A large percentage (60.7%) of our sample knew someone who had been seriously ill as a result of COVID-19, with these respondents perhaps unsurprisingly showing a greater willingness to vaccinate. Unlike previous studies on influenza vaccination, however [53], there was no significant association between belonging to a COVID-19 risk group and willingness to vaccinate.

This study was conducted relatively early on in the international vaccine rollout campaign. However in the six months following April 2021 approximately 700 million people worldwide received at least once vaccine [54], with a wide range of vaccines offered. In Study 2, we turn to vaccine willingness and preference for a booster in the third quarter of that year, in Thailand.

## 3. Study 2: Vaccine Willingness and Choice for a Further Vaccine in Thailand

### 3.1. Methods

#### 3.1.1. Participants

Between 4 and 19 October 2021, an established Thai survey company (‘Blue Eagle Eye’) collected data from across Thailand. Table 4 provides details of key demographics of our sample, including sex, age, education, occupation, and region from which the data were collected. Following a cognitive interview with participants to trial the questions, trained interviewers approached one in three pedestrians passing a randomly pre-determined point on regional shopping streets or near regional bus stations or local markets. Interviews took approximately six minutes. Interviewers were at least double vaccinated, used appropriate personal protective equipment (including facemasks and hand sanitizers), and maintained physical distance from interviewees at all times, in line with guidance from Thai national health authorities. During this time confirmed cumulative cases of COVID-19 rose from to 1.65 million to 1.81 million, with cumulative deaths from the coronavirus increasing from 17,100 to 18,407 [54]. However, at the time of study, there were no restrictions on movement besides a late evening curfew. If interviewers or interviewees displayed any of an expanded list of symptoms recognised by the Thai government as indicative of potential COVID-19 (e.g., fever, cough, shortness of breath), the interview was immediately terminated.

#### 3.1.2. Sample

We approached 1353 pedestrians who were passing our designated location; 200 of these turned away when they saw the interviewer so were not questioned. Of the 1153 people asked to participate, 89 refused, 12 were incomplete, and 1052 agreed to participate, leading to a response rate of 78% of those passing and 91% of those approached to participate. Those participating were given a small gift (pen) for their time. Data validation was performed by inspectors who independently observed 30% of the total interviews.

Participants were 51% female; ages ranged from 18 to 65 (M age = 39.91 (SD 12.99; national median for Thailand is 39 [32]). Responses were collected from 12 provinces, chosen to represent the five major regions of the country as a whole (290 respondents from Bangkok, 128 from the Bangkok neighbourhood, 153–166 respondents from regions in the Northeast, North, East (and Central) and South). Raw data and question items are deposited at https://osf.io/65gzp/ (accessed on 10 April 2022).

#### 3.1.3. Measures

Participants answered questions that included demographics, indicators of political trust and trust in other influencers, political party support, concern about infection, trust in vaccines and their efficacy, and reasons for being vaccinated. We report here associations between vaccine uptake and choice and political alignment and additional data about the main reasons for selection of different vaccines.

Demographic factors included age (in years), sex (1 = females, 0 = males), education (from elementary or lower to Masters degree or higher), occupation (9 groups, Table 4), and Province. *Household income* was ranked from very low to very high, in accordance with median monthly incomes for urban or rural regions (e.g., moderate was indicated as 30,000–50,000 THB for urban dweller, 25,000–40,000 THB as equivalent for rural/regional respondents). *Trust in government in vaccination* was indicated by a single item (*Do you trust the government to ensure that vaccines are safe and effective?* 4 points from *not at all* to *completely*). Political party choice was measured by the question *If there was a national general election tomorrow which party would you vote for?* with responses grouped into the five major parties and then further grouped into two groups: pro-government vs anti-government. Vaccine history was indicated by asking *Are you vaccinated* and if so *How many vaccines have you had?* Vaccine motivation was addressed by asking respondents if their main rationale for vaccination was to protect themselves, protect others, or whether this was a job requirement. Because most respondents were vaccinated, we asked *If you are offered your next dose of vaccine, or a booster dose, which would you prefer to have?* and allowed multiple answers. Options were Sinovac, AstraZeneca, Pfizer, Moderna, Johnson & Johnson, Sinopharm, Chulacov19, ‘any I can get first’ or ‘any that is free’. Participants could also indicate they were unsure or would not have any vaccine. They were then asked if their vaccine choice was based on a list of reasons, with participants given the option for multiple answers (*All the same to me, I had this already so want it again, I trust any government recommended vaccine, I trust Western vaccines more, I trust Chinese vaccines more, I trust Pfizer (Astrazeneca, Moderna, Johnson & Johnson, Sinopharm, ChulaCov19 more), work forces me to vaccinate, I will decide after the scheduled date*). Participants also rated each vaccine for its ability to (a) stop them from catching the virus, (b) stop them from spreading the virus, (c) stop them from getting seriously ill or dying (each a 7-point scale from *not at all* to *very much so*). They also indicated if they thought that the vaccine would have any specific side effects (*How worried are you about getting bad side effects from each of the vaccines?* on a scale from (1) not at all to (7) very much so).

#### 3.1.4. Analytic Strategy

First, we considered descriptive data regarding the first two doses of vaccine (doses taken, type of vaccine), motivation to vaccinate, tendency to trust the government in guaranteeing that vaccines are safe and effective, and trust in different professionals. We also describe participants’ beliefs in vaccine efficacy and side effects. We then present descriptive data about preference for a booster (third) dose. For this choice, we grouped vaccines into Eastern vaccines (Sinopharm, Sinovac, ChulaCov) and Western vaccines (AstraZeneca, Pfizer, Johnson & Johnson, Moderna).

Multivariate analyses then examined who had been vaccinated by political choice (pro vs. anti-government attitudes) and trust in government, controlling for age, sex, income, and education. For this, we conducted logistic regression, entering age, education, sex, and income in step 1 and trust in government and political views (pro vs. anti-government) in step 2. Logistic regressions were then conducted to examine associations between political preferences and booster vaccine choice. Predictors at step 1 were sex, age, education, and income. Individual vaccines were added at step 2. High levels of multi-collinearity between pro/anti-government and trust in government excluded the latter in this analysis. Finally, multinomial logistic regression analyses examined unadjusted and adjusted associations between political choice (predictor) and willingness to take any vaccine vs. none (reference group). Unadjusted odds ratios (OR) were calculated by entering age, education, income, and sex into the multinomial logistic regression in step 1 and political choice in step 2. Adjusted ORs (AOR) were calculated by adjusting for all predictor variables in the multinomial logistic regression simultaneously.

#### 3.1.5. Descriptive Data

The great majority of respondents had been vaccinated (*n* = 844, 80.2%), with most double vaccinated (60.6%), and a minority (0.8%) with their third dose. Respondents were most likely to have had a first dose of Sinovac (*n* = 340, 32%) or AstraZeneca (398, 37.8%), and AstraZeneca (*n*= 494, 47%) or Sinovac (96, 9.1%) as a second dose. Of those who had vaccinated (*n* = 844), respondents were most likely to say they had vaccinated mainly to protect themselves (*n* = 699, 82.8%) vs. to protect others (48, 5.7%), with a further 90 (10.7%) stating their job as the main reason for their vaccination. A small majority trusted the government in guaranteeing that vaccines are safe and effective (*n* = 573, 44.8% somewhat trusted, 9.7% completely trusted). There was higher trust towards medical practitioners (97.9% somewhat or completely), scientists (92.4%), religious leaders (67.7%), and influencers (e.g., actors or idols) (62.2%).

If offered a further vaccine, participants were most likely to indicate a preference for Pfizer (*n* = 402, 38.2%), Moderna (295, 28.0%), and AstraZeneca (194, 18.4%). Less preferred were Johnson & Johnson (*n* = 96, 9.1%), Sinopharm (80, 7.6%), Chulacov19 (30, 2.9%), and Sinovac (16, 1.5%). Trust in a specific vaccine was the main reason for this: 340 (32.3%) indicated that they trusted Pfizer the most, 247 (23.5%) Moderna, and 92 (8.7%) AstraZeneca. Respondents were most likely to indicate that Moderna and Pfizer would be most likely to stop them from catching COVID-19 (Ms 5.65/7 and 5.61, respectively), and were least likely to anticipate that Sinovac would prevent infection (M 3.46). Respondents were also most likely to see Pfizer and Moderna as the most effective for restricting spread (5.55 and 5.57) and Sinovac the least (3.48). Similarly, Pfizer and Moderna were rated the most likely to prevent serious illness (5.64, 5.65), ChulaCov the least (4.36). At the same time, concern about serious side effects was greatest for Moderna (4.71) and Pfizer (4.59), and lowest for Sinovac (4.01). Side effects were seen as positively correlated with perceived efficacy for each vaccine against serious illness (ranging from r = 0.15 to Chulacov19 (r = 0.48)).

For the choice of a next vaccine, we grouped vaccines into Eastern vaccines (Sinopharm, Sinovac, ChulaCov) and Western vaccines (Astrazeneca, Pfizer, Johnson & Johnson, Moderna). Sixty participants (5.7%,) chose Eastern-only vaccine while 60.6% (638) chose Western-only vaccines, and 5.3% (56) both Western and Eastern vaccines. Twenty-eight percent (298) chose no vaccine at all. Seven hundred and fifty-three (71.6%) respondents were willing to identify their political preferences. Of these, 540 (71.7%) could be identified as anti-government, and 213 (28.3%) pro-government. Participants who were anti-government were less likely to have been vaccinated than those who were pro-government (79.6% vs. 85.9%, χ2 (1) = 3.99 *p* = 0.046).

### 3.2. Multivariate Analyses

#### 3.2.1. Previous Vaccine and Political Choice

In Table 5, we examine the associations between vaccine uptake and demographics, trust that the government will ensure a safe and effective vaccine, and political party preference (coded as a party that supports opposes the government). As can be seen, those with greater trust in the government, the more educated, with higher income, males, and older respondents were significantly more likely to be vaccinated. Political choice was not significantly associated with the likelihood of having been vaccinated.

#### 3.2.2. Next Vaccine Preference and Political Choice

Table 6 examines associations between preference for a particular vaccine and political party choice, coded as anti or pro government, allowing for demographic factors. Higher scores indicate a stronger association with the government. In these logistic analyses, preference for AstraZeneca was associated with pro-government attitudes, and preference for Pfizer with anti-government views.

#### 3.2.3. Political Choice and Preferences for Eastern vs. Western Vaccines

Supplementary analyses (Appendix A Table A1) report associations between political preferences (anti vs. pro government) and choice of a Western vaccine, Eastern vaccine, or no vaccine at all, controlling for demographic factors. A pro-governmental political choice was significantly associated with a Western only vaccine choice compared to no vaccine. However, political choice was not associated with an Eastern-only vaccine choice, or with both Eastern and Western vaccines compared to no vaccine.

#### 3.2.4. Discussion: Study 2

Our respondents from Thailand also reported an association between government support and previous vaccination, reflecting the strong efforts made by the Thai government to promote their vaccination campaign. Overall, preference was for Pfizer and Moderna vaccines, as reported in other studies on vaccine acceptance in Thailand [18]. However, as was the case of Hungary, specific vaccines were more preferred by government supporters, and these reflected governmentally mandated choices (in this case, AstraZeneca). In contrast, Pfizer was preferred by respondents who opposed the main government supporting parties. In Thailand, the contrast was less clear between the choice of a broadly “Eastern” vs. “Western” vaccine than between a Western vaccine and no vaccine at all, with the preference of government supporters for Western vaccines a result of their willingness to accept the AstraZeneca vaccine, a vaccine strongly promoted by the government.

## 4. Discussion

Across the world, there is evidence of continued vaccine unease, with vaccine resistance having been identified as a major threat to global health even before the advent of COVID-19 [55]. Despite the importance of vaccination in the reduction of the spread of COVID-19 and in the limiting of mortality and morbidity from the virus [56], vaccine rollouts have been embroiled in uncertainty, rumours, and conspiracy theories [6]. Vaccine willingness can change rapidly as the reputation of different vaccines varies over time, as evident in the loss of trust in the AstraZeneca vaccine across several European countries, the US [57], and Thailand [6], and in the questioning of the efficacy of the Sinopharm vaccination in Hungary [58]. All this means that not only vaccine willingness, but also the choice of vaccine has become a highly significant area for study.

The politicisation of vaccine roll outs [13], and disputes over the safety or efficacy of specific vaccines [14], have contributed to high national rates of scepticism in several cultures. In two studies conducted in countries where vaccination for COVID-19 has been closely associated with a strong political divide or ongoing anti-government protests and unrest, we find that political preference and trust have important implications for both willingness to take a vaccine and, significantly, the choice of vaccine. In these countries, specific vaccines promoted by national authorities were less likely to be chosen by those who supported political parties opposing the ruling government. As our data from Thailand also show, some vaccines were partly rejected as they were viewed as less efficacious against infection prevention and serious disease, consistent with findings reported from physicians in that country [21]. In both our studies, males and older respondents were more likely to express willingness to vaccinate, in line with data collected elsewhere [46,59]. Consistent with previous surveys, education and income were positively associated with willingness to take the COVID-19 vaccine [14], consonant with cross-cultural evidence suggesting the more numerate are less suspect to COVID-19 misinformation [60].

### 4.1. Limitations

We recognise both strengths and limitations to both our studies. The availability of specific vaccines in different countries limited the options open to our study participants, and thus our understanding of perceptions towards a wider range of vaccines. Because of the speed of the evolving vaccination situation, the survey companies involved expedited data collection within a short time period. Both our samples were cross-sectional, and we were therefore not able to assess predictors of vaccine willingness over time. In both studies, a proportion of respondents did not indicate a named party, limiting our analyses of specific political groupings in each analysis. Further, although we used different methods for data collection in each study, neither of our samples could be claimed to be random. In Hungary, we made use of an online survey to collect a sample as representative of the general population as possible. Although this recruitment method is widely used and may be important, particularly for the collection of time-sensitive data during a pandemic [61], respondents in the sample pool may not be more widely representative of the population, and quota sampling has limitations for ascertaining accurate response rates [47,62]. In Hungary, we purposefully targeted the unvaccinated, and assessed only behavioural intentions to be vaccinated, rather than actual vaccination behaviour. Although intentions and behaviour are strongly linked here [63], attitudes towards any vaccination may vary as populations acquire further experience with the vaccination rollout. In Thailand, the use of on-street sampling from a wide range of locations across the country meant we were able to more appropriately compute response rates, but we cannot be sure that those with particular characteristics were being represented (e.g., those reluctant to go outside because of the virus). Finally, we acknowledge that political divisions in a country do not necessarily mean that there will be high vaccine hesitation or rejection by particular sub-groups. While the literature on political trust suggests this to be an important aspect in vaccine uptake, we studied two cultures where there was a selection of vaccines and a relatively large amount of vaccine available in the country. In other cultures, the focus may be primarily on issues of obtainability rather than choice. For example, in a politically divided India, where vaccination willingness was relatively high, political discussions focused mainly on concerns about vaccine availability [64]. Further, in other countries, where political choice is limited or non-existent, such a focus for vaccine research may be impractical, and the impact of such preferences on vaccine uptake or selection minimal. This suggests caution when generalising our studies to some other countries.

### 4.2. Implications

Despite the above, we believe our findings have significant implications for those aiming to encourage vaccine uptake. As noted above, health crises can destabilise a sense of shared national identity [24]. In Thailand, concern about the Sinovac vaccine has contributed towards increased political tensions [65]. Any initial sense of commonalities may be challenged by societal divisions, with this most evident amongst the most disadvantaged groups in a society [66]. There is thus a need to continue to promote a shared sense of purpose and identity throughout a pandemic [67]. Our results show that vaccine campaigns need to recognise the role of political loyalties not only in vaccine willingness, but in vaccine choice, and should acknowledge the interplay between trust, ideology, and different regulators. This may become particularly important as different combinations of vaccines are used across doses. Political rhetoric influences public action via social identification [67]. National leaders need to provide clear and consistent recommendations [27]. Culturally specific rhetoric is likely to have an important impact on the measures used to manage a pandemic, including vaccination drives. Given the difficulties in gaining trust across a country during a pandemic, it may be particularly important to draw upon trusted sources, such as local clinicians, in order to assuage doubts and suspicions [8]. Some populations, such as Roma across Europe, are likely to distrust top-down messages from national health-care systems [68]. Localised campaigns can be accompanied by broader initiatives which can combine text messaging with a range of social media to provide nuanced, value-laden messages. Offering vaccines within specific and convenient settings can help reach difficult to access populations and increase trust [69]. Different platforms can be used to target specific audiences [70], e.g., those political groups with specific concerns about a vaccine, or vaccination in general. Where appropriate, specific cultural communities can be targeted, drawing on awareness of specific values within a culture or sub-culture [70,71]. In this case, specific groups may benefit from group-based health promotion, as has been evident amongst Cambodian and Myanmar migrants in Thailand [65]. Evidence suggests that vaccine campaigns should combine an emphasis on safety with a celebration of the gains that arise from the normalisation of social activities [8]. The efficacy of any such messaging can then be tracked via experimental studies and measured against large-scale national household longitudinal studies as COVID-19 continues to evolve and provide challenges for vaccination programmes across the globe.

### 4.3. Conclusions

The development of a range of vaccines for COVID-19 has been accompanied by a substantial body of research examining the drivers of vaccine acceptance, hesitancy, and refusal. However, while a number of studies have identified the role of politics in vaccine willingness, less attention has been paid to the influence of political preferences on the choice of specific vaccines. Using data from two very different, politically divided countries (Hungary and Thailand), we find that support of the governing party was associated with preference for a vaccine promoted by that government, even at a time when this vaccine was being questioned in the media for its efficacy or safety. In contrast, opposition to the ruling party was associated with preferences for other MRNA vaccines. Understanding this association between political factors and vaccine choices is essential for public health professionals keen to reach specific audiences and to address particular anxieties over vaccine safety and efficacy.

## Figures and Tables

**Table 1:** Study 1, Hungary: Sample characteristics, *n* = 1130.

	Frequency	Percent	Mean	SD
Age			50.53	15.92
Sex (female)	618	54.7		
Education (highest level attained)				
- Primary	44	4.8		
- Secondary	647	57.3		
- College/BA	276	24.4		
- Masters or Doctoral level	163	14.4		
Region				
- Central	336	33.9		
- Western	288	29.1		
- Eastern	367	37.0		
Previously had COVID (yes)	124	11.1		
Knew someone previously ill from COVID (yes)	687	60.7		
Self-rated health (4 point, low (1) to high (4))			2.71	0.77
Risk group membership (yes)	469	41.5		
Support for FIDESZ or other parties				
- FIDESZ	173	27.0		
- Other parties	468	73.0		
Political positioning				
- Left wing/centre	300	46.8		
- Right wing	341	53.2		
Political party supported				
- FIDESZ	173	19.1		
- Jobbik	122	13.5		
- Democratic Coalition	105	11.6		
- Opposition Alliance	72	8.0		
- Momentum	59	6.5		
- Two tailed dogs	32	3.5		
- Right list	25	2.8		
- Greens	17	1.9		
- Our homeland	14	1.5		
- Socialist	14	1.5		
- Christian democrat	7	0.8		
- Communist	1	0.1		
- No preference	78	8.6		
- Won’t say or Don’t know	186	20.5		

**Table 2:** Path analyses of vaccine willingness by political grouping (Study 1, Hungary).

	Government Approved Vaccine			EMA Approved Vaccine		
**1. FIDESZ vs. other Parties**	*p*	β	b	*p*	β	B
Sex (male = 1, female = 0)	<0.001	0.13	0.37 ***	0.19	0.12	0.33
Age	0.19	0	0	0.003	0.09	0.01 **
Education	0.01	0.08	0.14 **	<0.001	0.2	0.33 ***
Risk group (yes)	0.08	0.06	0.17	0.03	0.07	0.20 *
Covid personally infected (no)	0.55	−0.02	−0.08	0.86	−0.01	−0.02
Covid others infected (no)	<0.001	−0.09	−0.27 ***	0.003	−0.09	−0.27 **
Self rated health	0.98	0	0	0.004	−0.07	−0.13 **
FIDESZ (1) vs. others (0)	<0.001	0.37	1.21 ***	0.02	0.08	0.27 *
**2. Right vs. centre/left political party**						
Sex (male = 1, female = 0)	<0.001	0.1	0.29 ***	<0.001	0.1	0.29 ***
Age	0.07	0.06	0.01	0.01	0.09	0.01 **
Education	0.004	0.09	0.15 **	<0.001	0.17	0.29 ***
Risk group (yes)	0.14	0.05	0.15	0.04	0.07	0.20 *
Covid personally infected (no)	0.51	−0.02	−0.09	0.92	0	−0.01
Covid others infected (no)	0.001	−0.09	−0.28 **	<0.001	−0.1	−0.27 **
Self rated health	0.94	0	0.01	0.03	−0.07	−0.13 *
Right party (1) vs. centre or Left party (0)	<0.001	0.25	0.73 ***	0.14	−0.05	−0.16
**3. Political leaning (percentage scale)**						
Sex (male = 1, female = 0)	0.01	0.08	0.28 ***	0	0.11	0.31 ***
Age	0.06	0.06	0.01	0.01	0.09	0.01 **
Education	0.27	0.03	0.06	0	0.18	0.31 ***
Risk group (yes)	0.07	0.03	0.18	0.04	0.07	0.20 *
Covid personally infected (no)	0.55	−0.02	−0.08	0.09	0	−0.02
Covid others infected (no)	0	−0.1	−0.29 ***	0	−0.09	−0.27 ***
Self rated health	0.96	0	0	0.02	−0.07	−0.13 *
Political tendency	0	0.22	0.02 ***	0.24	−0.03	0

Note: *** *p* < 0.001; ** *p* < 0.01 * *p* < 0.05. b—unstandardized estimates, β—standardized estimates.

**Table 3:** Vaccine preference and political affiliation in Hungary (Study 1: logistic regression).

	Political Grouping of Right vs. Left			Political Grouping of FIDESZ vs. Other Parties			Political Tendency (Left-Right Percentage)		
Background Variables	b	Wald	*p*	b	Wald	*p*	b	Wald	*p*
Sex (male = 1)	−0.31	2.81	0.09	−0.78 ***	13.07	0	1.09	0.03	0.39
Age	−0.02 **	12.06	0.001	−0.004	0.31	0.58	−0.12 **	−0.1	0.005
Education	−0.51 ***	18.23	0	−0.38 **	6.91	0.01	0.69	0.03	0.37
Risk group (yes)	0.1	0.24	0.62	−0.03	0.01	0.92	−1.87	−0.05	0.2
Covid: personally infected (yes)	0.52	3.13	0.08	0.39	1.18	0.28	2.2	0.03	0.26
Covid: others infected (yes)	−0.04	0.05	0.82	0.1	0.23	0.64	0.5	0.01	0.7
Self Rated Health	−0.03	0.05	0.82	0.04	0.05	0.82	0.25	0.01	0.78
**Vaccine types**									
Johnson/Johnson	−0.3	1.73	0.19	0.03	0.01	0.92	−2.43	−0.05	0.12
Moderna	−0.66 **	8.88	0.003	−0.93 **	10.53	0.001	−3.10 *	−0.07	0.05
AstraZeneca	0.14	0.19	0.66	−0.28	0.53	0.47	0.79	0.01	0.73
Pfizer	−0.46 *	4.89	0.03	0.01	0.003	0.96	−2.93 *	−0.07	0.04
Sinopharm	1.28 ***	16.75	0	1.58 ***	30.76	0	11.23 ***	0.18	0
Sputnik	0.83 **	11.85	0.001	1.37 ***	31	0	3.42 *	0.07	0.03

Note: *** *p* < 0.001; ** *p* < 0.01; * *p* < 0.05. b—unstandardized estimates *p*—significance of Wald test.

**Table 4:** Participants in Thailand (*n* = 1052, Study 2).

Variable	Number	Percent	Mean	SD
Sex (female)	537	51		
Age			39.01	12.99
Education (highest)				
- Elementary or lower	134	12.7		
- High school	386	36.7		
- Bachelor	515	49.0		
- Masters and above	17	1.6		
Occupation				
- Business owner	102	9.7		
- Government worker	115	10.9		
- Company worker	390	37.1		
- contract worker/part-time/freelance	241	22.9		
- Unemployed	26	2.5		
- Student	89	8.5		
- Retired	16	1.5		
- Housewife/husband	73	6.9		
Province				
- Bangkok	290	27.6		
- Bangkok Metropolitan	128	12.2		
- Northern	166	15.8		
- North Eastern	156	14.8		
- Central	158	15.0		
- Southern	154	14.6		
Household income				
- Very high	183	17.4		
- High	122	11.6		
- Medium	451	42.9		
- Low	156	14.8		
- Very low	140	13.3		

**Table 5:** Study 2 (Thailand): Vaccine uptake by political choice and trust in government (logistic regression).

	B	S.E.	Wald	Sig.	Exp (B)	95% Confidence Interval for Exp (B)
Sex (male = 0 female = 1)	−0.46 *	0.20	5.18	0.02	0.63	0.43, 0.94
Age	0.03 ***	0.01	12.20	0.000	1.03	1.01, 1.05
Education	0.93 ***	0.16	34.77	0.000	2.54	1.86, 3.46
Income	0.13 ***	0.04	12.90	0.000	1.14	1.06, 1.23
Pro vs. Anti-government Against (0) or for (1)	0.03	0.26	0.01	0.92	1.03	0.62, 1.70
Trust government vaccines are safe and effective	0.36 **	0.12	9.50	0.002	1.43	1.14, 1.80

Note: *** *p* < 0.001; ** *p* < 0.01 * *p* < 0.05. B is unstandardized estimate, with significance for Wald test; Exp (B) represents odds ratio.

**Table 6:** Political choice (anti vs. pro government) by individual vaccines.

	B	S.E.	Wald	Sig.	Exp (B)	95% Confidence Interval for Exp (B)
Sex (male = 0 female = 1)	0.31	0.17	3.06	0.08	1.36	0.96, 1.91
Age	0.03 ***	0.01	21.86	0.00	1.03	1.02, 1.05
Education	0.37 **	0.13	8.00	0.005	1.45	1.12, 1.88
Income	−0.01	0.08	0.01	0.94	0.99	0.85, 1.16
Sinovac	0.11	0.70	0.02	0.88	1.11	0.28, 4.40
Astrazeneca	0.45 *	0.21	4.44	0.035	1.57	1.03, 2.37
Pfizer	−0.59 **	0.20	9.17	0.002	0.55	0.38, 0.81
Moderna	0.03	0.21	0.02	0.89	1.03	0.68, 1.56
Johnson & Johnson	−0.41	0.35	1.31	0.25	0.67	0.33, 1.33
Sinopharm	0.39	0.29	1.82	0.18	1.48	0.84, 2.59
Chula	0.35	0.50	0.50	0.48	1.43	0.53, 3.83

Note: *** *p* < 0.001; ** *p* < 0.01 * *p* < 0.05. B is unstandardized estimate, with significance for Wald test; Exp (B) represents odds ratio.

## Data Availability

Data can be found at https://osf.io/65gzp/ (accessed on 10 April 2022).

## Appendix A

**Table A1:** Multinomial regression on willingness to accept Western, Eastern, or either vaccine vs. no vaccine, by political choice.

	B	Std. Error	Wald	Sig.	Exp (B)	95% Confidence Interval Exp (B)
**East-Vaccine only***n* = 60 5.7% (reference group = no vaccine)						
Sex (male = 0 female = 1)	−0.43	0.32	1.78	0.18	0.65	0.35, 1.22
Age	0.03 *	0.01	6.08	0.01	1.03	1.01, 1.06
Education	0.71 **	0.25	7.94	0.005	2.02	1.24, 3.30
Income	0.03	0.14	0.04	0.84	1.03	0.78, 1.36
Anti-government (0) vs. Pro (1)	0.04	0.34	0.01	0.90	1.04	0.54, 2.01
**West-Vaccine only***n* = 638 60.6% (reference group = no vaccine)						
Sex (male = 0 female = 1)	−0.24	0.17	1.98	0.16	0.78	0.56, 1.10
Age	0.01	0.01	1.75	0.19	1.10	1.00, 1.02
Education	0.12	0.13	0.93	0.34	1.13	0.88, 1.45
Income	0.03	0.07	0.16	0.69	1.03	0.89, 1.19
Anti-government (0) vs. Pro (1)	0.61 **	0.19	10.64	0.001	1.84	1.28, 2.66
**Both West and East vaccine***n* = 56 5.3% (reference group = no vaccine)						
Sex (male = 0 female = 1)	−0.37	0.38	0.96	0.33	0.69	0.33, 1.45
Age	0.03 *	0.02	4.33	0.04	1.03	1.00, 1.07
Education	0.39	0.28	1.92	0.17	1.48	0.85, 2.57
Income	−0.10	0.17	0.33	0.56	0.91	0.65, 1.26
Anti-government (0) vs. Pro (1)	0.03	0.40	0.004	0.950	1.03	0.47, 2.23

Note: ** *p* < 0.01 * *p* < 0.05.
